# Reliability and Validity of Emotrics in the Assessment of Facial Palsy

**DOI:** 10.3390/jpm13071135

**Published:** 2023-07-13

**Authors:** Min Gi Kim, Cho Rong Bae, Tae Suk Oh, Sung Jong Park, Jae Mok Jeong, Dae Yul Kim

**Affiliations:** 1 Department of Rehabilitation Medicine, Asan Medical Center, University of Ulsan College of Medicine, Seoul 05505, Republic of Korea; d190480@amc.seoul.kr (M.G.K.); june7258318@hanmail.net (C.R.B.); boyparksj@naver.com (S.J.P.); jaemoke@amc.seoul.kr (J.M.J.); 2 Department of Plastic and Reconstructive Surgery, Asan Medical Center, University of Ulsan College of Medicine, Seoul 05505, Republic of Korea; tasuko@amc.seoul.kr

**Keywords:** facial palsy, automated evaluation, emotrics, interrater reliability, House–Brackmann facial grading system

## Abstract

The globally accepted evaluation method for facial palsy is the House–Brackmann facial grading system; however, it does not reflect minute changes. Several methods have been attempted, but there is no universally accepted evaluation method that is non-time-consuming and quantitative. Recently, Emotrics, a two-dimensional analysis that incorporates machine-learning techniques, has been used in various clinical fields. However, its reliability and validity have not yet been determined. Therefore, this study aimed to examine and establish the reliability and validity of Emotrics. All patients had previously received speech therapy for facial palsy at our hospital between January and November 2022. In speech therapy at our hospital, Emotrics was routinely used to measure the state of the patient’s facial palsy. A frame was created to standardize and overcome the limitation of the two-dimensional analysis. Interrater, intrarater, and intrasubject reliability were evaluated with intraclass correlation coefficients (ICC) by measuring the indicators that reflect eye and mouth functions. Validity was evaluated using Spearman’s correlation for each Emotrics parameter and the House–Brackmann facial grading system. A total of 23 patients were included in this study. For all parameters, there was significant interrater and intrarater reliability (ICC, 0.61 to 0.99). Intrasubject reliability showed significant reliability in most parameters (ICC, 0.68 to 0.88). Validity showed a significant correlation in two parameters (*p*-value < 0.001). This single-center study suggests that Emotrics could be a quantitative and efficient facial-palsy evaluation method with good reliability. Therefore, Emotrics is expected to play a key role in assessing facial palsy and in monitoring treatment effects more accurately and precisely.

## 1. Introduction

Facial palsy is a neurogenic disorder involving the movement of facial muscles, potentially resulting in restricted facial expressions, lower quality of life, and depression [1,2]. In severe cases, corneal keratitis and blindness may occur due to incomplete eye closure. Articulation disorders and dysphagia may also occur due to the restriction of lip sealing [3,4]. Among the evaluation methods for facial palsy, the House–Brackmann facial grading system (the HB grades) is a clinician-graded scoring system that enables intuitive evaluation with high interrater reliability and is used worldwide [5]. However, this system roughly categorizes facial palsy into six stages, thus representing a major limitation as it cannot reflect minute changes [6].

Various methods utilizing two-dimensional (2D), three-dimensional (3D), and motion analysis have been developed to detect minute changes, although, there is currently no worldwide objective evaluation method [6,7]. Three-dimensional analysis and motion analysis require expensive special equipment, and the results needed complicated calculations. Therefore, accessibility is limited [2,8]. Additionally, manual measurements, like the eFACE program developed by Banks and colleagues, are highly time-consuming to obtain results [9,10]. However, a program developed by the Massachusetts Eye and Ear Institute called Emotrics was recently presented as an automatic evaluation method that combines machine-learning technology with 2D analysis [11]. This can be a complementary method used in a clinical setting for facial palsy as it can be evaluated efficiently by taking pictures.

Emotrics possesses a significant advantage in automatically evaluating the differences in landmark positions between two images. It has been utilized in assessing the effectiveness of various plastic surgeries and rehabilitation treatments [9,12,13,14]. However, since it was not originally designed for the purpose of the facial palsy severity scale, there is insufficient evidence to support the reliability and validity of its automatic evaluation, particularly when compared to manual evaluation methods or the HB grades. Nevertheless, as minimal head rotation allows for a side-to-side comparison, we have planned a study on the facial palsy evaluation method using this program. On the other hand, eFACE, a manual measurement method, reportedly has remarkable interrater, intrarater, intrasubject reliability [10,15]. Therefore, this study aims to address this research gap and establish fundamental evidence by examining the reliability and validity of Emotrics as a valuable tool for automatically assessing facial palsy. The findings of this study are anticipated to contribute to improved quantitative and efficient measurements of facial palsy.

## 2. Materials and Methods

### 2.1. Participants

We obtained approval for this study from the Institutional Review Board of Asan Medical Center (No. 2022-1567; Seoul, Republic of Korea) prior to data collection. This study was a single-center retrospective study, targeting patients who received speech therapy for facial palsy at the Department of Rehabilitation Medicine. The inclusion criteria included patients with either central or peripheral facial palsy, who were diagnosed in our department, who were able to follow instructions, and who could cooperate with the photo shoot. The exclusion criteria were those with congenital orofacial deformities that made it difficult to determine the degree of facial palsy, those with other known facial disorders or orofacial pain, and those with bilateral involvement.

### 2.2. Interventions

**Figure 1:**
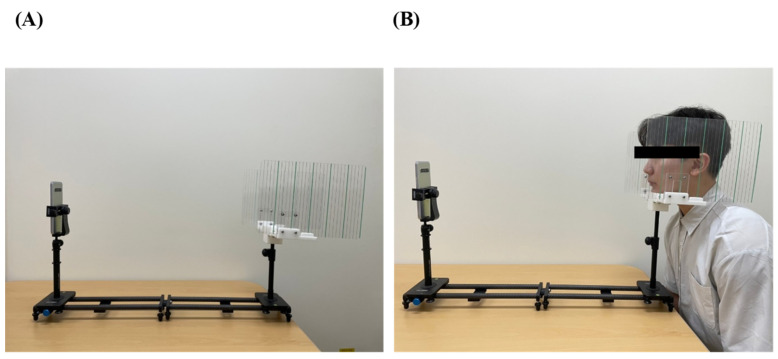
Composition of the evaluation frame. (**A**) The frame was composed of a chin rest on one side and phone holder on the other. The distance was fixed with a bar. On the side with the chin rest, markings were placed bilaterally to confirm a neutral position. (**B**) The heights of the chin rest and phone holder could each be individually adjusted for the patient.

The speech therapy conducted on facial palsy patients in our department aimed to improve weakened facial muscle through sensory and motor stimulation of the mandibular, labial, lingual, buccal, and ocular muscles. At each treatment session, facial palsy was initially evaluated by a speech–language pathologist using Emotrics and HB grades. The utilization protocol for the program is as follows: A frame was created to standardize the images. The frame had a face holder at one end and a camera holder at the other, with a distance-control bar located between them (Figure 1). An iPhone 12 Pro camera was used for shooting. The picture was taken using the default settings, with a focal length of 26 mm and without any magnification. The distance was fixed at 70 cm between the face and the camera. To minimize facial rotation, the exposure of both ears was equalized by aligning the placement between bilateral external auditory meatuses and markings fixed to the frame. To improve the program’s ability to recognize eyebrow landmarks, the hair was pulled back so that the forehead was clear. In order to enhance the recognition rate of lip landmarks, facial hair was removed before the test. The face was placed in the center of the photograph, with the camera positioned parallel to the face to standardize and minimize peripheral distortion caused by a wide-angle lens. The shooting protocol is summarized below.

- Distance is fixed (70 cm).
- Do not rotate the head (tilt is acceptable).
- Clean the forehead.
- Perform facial hair removal prior to the evaluation.
- Place the face in the middle of the photograph.
- Camera should be placed parallel to the face.

With the above settings, a total of three shots per session were taken of gently opened or closed eyes or smile. This study analyzed the pictures captured during the first speech therapy session and during the second follow-up session conducted within a week.

### 2.3. Baseline Characteristics

Age, gender, and facial-palsy-related characteristics of the patients were collected through the electrical medical-record system. The laterality, type (central or peripheral), and etiology of the facial palsy, as well as the impairments of the eyes and mouth assessed by an experienced speech–language pathologist, were summarized.

### 2.4. Emotrics Parameters

**Figure 2:**
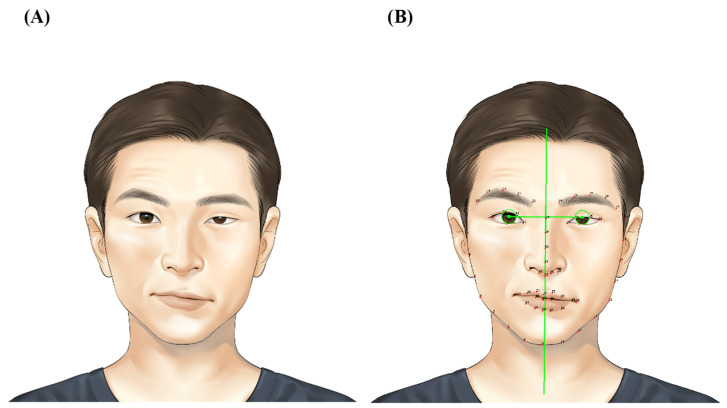
Placement of landmarks by Emotrics. (**A**) Classic peripheral type of facial-palsy patient was described. (**B**) Emotrics automatically placed landmarks according to machine-learning technology. If a picture was entered into the program, the results of the parameters were automatically calculated.

When analyzing the photographs using the Emotrics program, the program automatically displayed 68 landmarks and performed a quantitative analysis of facial symmetry and function. This analysis included the assessment of symmetry in the eyebrows, width of the palpebral fissure (PF), degree of movement, and symmetry of the lips during smiling based on the distances between the landmarks (Figure 2). However, owing to the inherent characteristics of machine learning, multiple automatic evaluations of the same image did not consistently yield identical results. Therefore, to validate the precision of the automatic assessment, a comparative analysis was conducted between the automatic assessment and the in-person assessment, thereby demonstrating interrater reliability in this research. Moreover, to establish the test–retest reliability, multiple automatic assessments were compared, as demonstrated by the intrarater reliability in this study. The parameters used to analyze the results included brow height (BH) and PF related to the eyes, and commissure excursion (CE), smile angle (SA), and dental show (DS) related to the mouth. The affected–unaffected value (A-U), which expressed the difference between the affected and unaffected sides of BH in the resting state as a ratio, was defined as (BH of affected side—BH of unaffected side)/BH of the unaffected side. The difference between the corresponding value in the resting state and eye-closing state (r-c) was defined as BH_r-c. PF_A-U and PF_r-c were calculated similarly. In addition, the CE, SA, and DS related to the mouth confirmed the difference between the corresponding value in the resting state and smiling state (r-s), not the r-c value. Values were defined as CE_A-U, CE_r-s, SA_A-U, SA_r-s, DS_A-U, and DS_r-s, as corresponding values.

### 2.5. Statistical Analysis

Statistical analysis was conducted using IBM SPSS Statistics for Windows, version 25 (IBM Corp., Armonk, NY, USA). The reliability of the Emotrics parameters were evaluated using the intraclass correlation coefficient (ICC). Interrater reliability was assessed between the automatic and in-person assessment results. It was calculated using the average of the values, evaluated twice automatically and measured twice manually by an experienced physician who majored in neurorehabilitation, with the photo taken during the first session using ICC (2,1). For intrarater reliability of automatic assessments, the values from the first session that were automatically measured twice were compared using ICC (2,1). This is because the results were slightly different each time, even if the same photo was repeatedly evaluated automatically. For intrasubject reliability, automatically measured values from the first and second sessions were compared using ICC (2,1). The 95% confidence interval of each ICC value was calculated, and the degree was specified according to the value: poor was below 0.20, fair was between 0.21 and 0.40, moderate was between 0.41 and 0.60, good was between 0.61 and 0.80, and very good was between 0.81 and 1.00. Validity was evaluated using Spearman’s correlation coefficient for each Emotrics parameter and the HB grades. Statistical significance was set at *p* < 0.05.

## 3. Results

### 3.1. Baseline Characteristics

Between January and November 2022, 796 patients were diagnosed with facial palsy at our hospital. It is important to note that all participants enrolled in this study were exclusively from the East Asian racial group, specifically Korean. Among them, 23 received two or more speech therapy sessions, and an Emotrics evaluation was performed correctly (Table 1). The average age was 49.4 years. Gender and laterality of the involved side were similarly distributed. Peripheral facial palsy was more common than the central type. As for severity, 10 patients had HB grade I on the eye side, and 20 had HB grade IV or V on the mouth side.

### 3.2. Facial Palsy Parameters

In all 10 Emotrics parameters, interrater and intrarater reliability showed significant consistency, with most maintaining an ICC value of at least 0.80 except one interrater reliability of BH_r-c (ICC = 0.61 [95% CI, 0.02–0.84]) (Table 2). Furthermore, the A-U value showed higher reliability than the r-c or r-s values. Although intrasubject reliability showed significant reliability in many parameters, it showed a lower value compared to other types of reliability. Intrasubject reliability of BH_A-U, PF_A-U, CE_A-U, CE_r-s, SA_A-U, and SA_r-s showed ICC values of at least 0.68. On the other hand, the other parameters did not show meaningful ICC values. When comparing Emotrics parameters with the HB grades to evaluate validity, only BH_r-c and PF_r-c showed significant correlations (Table 3).

## 4. Discussion

Facial palsy, which can lower quality of life and cause psychiatric distress, can be divided into the peripheral type and central type according to the location of the damaged nerve. To differentiate between the subtypes, involvement of the forehead motor function, innervated by facial nerves from the bilateral motor cortex, is important. In the case of eye closure, part of the orbicularis oculi is bilaterally innervated, which can lead to mild impairment even in the central type [16]. Therefore, Emotrics, an automatic and quantitative measurement method for eye and mouth dysfunction, regardless of lesion location, is important for facial palsy.

Even though the original purpose of this program was not to assess the severity of facial palsy but simply to calculate facial measurements, we performed this study to demonstrate the feasibility of utilizing the program for the facial palsy severity scale [11]. The emphasis was placed on minimizing facial rotation to ensure the symmetrical positioning of landmarks on both sides to enable the comparison of facial parameters between both sides. In this study, the reliability of Emotrics for facial palsy patients was subdivided into interrater, intrarater, and intrasubject evaluations [2]. To assess the ability of automatic measurements, the automatically and manually measured data were compared with interrater reliability. Moreover, to overcome the limitations of the 2D analysis, all pictures were taken under the same conditions using a new frame designed for this program [6]. To minimize the peripheral distortion that inevitably occurs due to the characteristics of wide-angle lenses, the face was placed centrally. Lastly, to standardize the positional relationship between the 2D plane and the 3D landmarks, the camera was placed parallel to the face [17]. As a result, most parameters representing the functions of the eyes and mouth showed very good reliability. Although Emotrics will show a progressively better assessment function due to the nature of the program implemented through machine learning, the current system still produced sufficiently meaningful results [11,18].

A previous study confirmed the improvement of BH and PF using Emotrics, displaying improved eye function after plastic surgery on the eyelids that did not close in facial palsy [9]. Previous studies have also shown improvements in SA, CE, and DS using this program, representing oral functions, as well as additional progress in other indicators after rehabilitation treatment or plastic surgery that enabled spontaneous smiling [12,13,14]. Based on these studies, Emotrics parameters are well-reflected by clinical improvements. It is a meaningful evaluation method with actual clinical applications. Thus, this study presents evidence for the reliability and validity of the program, which had not previously been established.

Comparing the ICC values, the high ICC of interrater reliability implies that there was not a substantial difference between the automatically and manually obtained results. Therefore, the results automatically derived through machine learning represented a meaningful result. Additionally, the A-U value evaluated by the resting status showed higher reliability than the r-c and r-s values, which evaluate the difference between the resting status and the closed-eye or smiling statuses. Moreover, the inter- and intrarater reliability evaluated with the same picture showed higher consistency than the intrasubject reliability evaluated with different pictures, suggesting that the result can be different every time a new picture is taken. This is a result of the limitations of the previously recognized 2D analysis; hence, it is necessary to consider a more precise shooting protocol. However, most reliability indicators displayed good to very good reliability, and the result of this program is comparable to other, more time-consuming tools [2,10,15]. Therefore, Emotrics has the strength of obtaining meaningful results immediately through automatic evaluations.

Despite the limitation of being unable to detect subtle changes, the HB grades can be deemed as the gold standard for evaluating facial palsy due to their global acceptance. Upon comparing the Spearman’s correlation coefficient of the results, no significant correlations were found between most parameters and the HB grades (Table 3). Even though BH_r-c and PF_r-c showed a remarkable correlation, those parameters did not exhibit a significant intrasubject reliability value. When examining the distribution of the patient groups, for HB grade of the eye, patients with mild symptoms (classified as HB grade I) accounted for half of the patients. Additionally, for HB grade of the mouth, patients with severe symptoms, such as those with HB grades IV and V, comprised the majority. This is because of the considerable number of central-type patients enrolled in this study. Therefore, our results show a skewed distribution, with increased involvement of the mouth rather than the eyes. In fact, the HB grades of the eye of central-type patients who were enrolled were all either below or equal to grade II. When a subgroup analysis was performed on 14 people, excluding central-type patients, the correlation coefficient increased overall compared to the analysis of the original population, and the *p*-value was also lower (Table 4). This finding suggests that, in a patient population with a more even distribution, a stronger correlation may be observed.

This study has some limitations. First, it is important to note that the findings are derived from a pilot study conducted with a small and unevenly distributed patient group. Therefore, to assess the validity of the program, a larger-scale study with a more representative and well-distributed population is required. Furthermore, it is important to acknowledge that Emotrics has historically targeted the Caucasian population [11]. However, this study exclusively targeted individuals belonging to the East Asian racial group, characterized by relatively darker skin tones. As a result, there may be a decrease in the accuracy of landmark placement, leading to potential bias. Therefore, further studies targeting diverse racial groups would be beneficial. Second, to overcome the limitations of the 2D analysis, we developed a shooting protocol that involved the use of a frame. However, it is important to note that this approach may limit accessibility for clinicians. Hence, conducting additional research comparing the photographs captured with and without the use of the frame would further enhance accessibility. Third, based on the findings of that study, it may be possible to establish a cutoff value for each HB grade by selecting the specific parameters that can be correlated with the HB grades. This would provide a more standardized and clinically applicable interpretation of the Emotrics results. Fourth, it should be acknowledged that this assessment tool has limitations in capturing synkinesis resulting from facial nerve reinnervation, which warrants further research in additional evaluations [5]. Lastly, exploring additional evaluation methods that encompass nonverbal communication cues such as smiling may enhance the comprehensiveness of the facial-expression assessment [19].

## 5. Conclusions

Most Emotrics parameters are statistically significant and show good to very good interrater, intrarater, and intrasubject reliability. This single-center study suggests that this program could identify changes in the severity of facial palsy more delicately than the conventional method. Overall, Emotrics, a quantitative and automatic evaluation method for facial palsy, can play an important role in assessing facial palsy and monitoring the effectiveness of the treatments.

## Figures and Tables

**Table 1:** Baseline characteristics of the patients.

Characteristics	Total Patients (*n* = 23)
Age, years	49.4 ± 17.9
Sex	
Female	11/23 (47.8%)
Male	12/23 (52.2%)
Facial-palsy-related feature	
Laterality	
Right	12/23 (52.2%)
Left	11/23 (47.8%)
Type	
Central type	9/23 (39.1%)
Peripheral type	14/23 (60.9%)
Etiology	
Bell’s palsy	4/23 (17.4%)
Cerebrovascular accident	6/23 (26.1%)
Brain tumor	10/23 (43.5%)
Trauma	2/23 (8.7%)
Unidentified	1/23 (4.3%)
House–Brackmann facial palsy scale	
Eye	
1	10/23 (43.5%)
2	4/23 (17.4%)
3	1/23 (4.3%)
4	4/23 (17.4%)
5	3/23 (13.0%)
6	1/23 (4.3%)
Mouth	
2	3/23 (13.0%)
4	10/23 (43.5%)
5	10/23 (43.5%)

Values are n/N (%) or mean ± standard deviation.

**Table 2:** Interrater, intrarater, and intrasubject reliability of multiple Emotrics parameters.

Characteristics	Interrater Reliability	Intrarater Reliability	Intrasubject Reliability
BH_A-U	0.99 CI (0.98, 0.99) *	0.97 CI (0.94, 0.99) *	0.76 CI (0.41, 0.90) *
BH_r-c	0.61 CI (0.02, 0.84) *	0.95 CI (0.88, 0.98) *	−0.82 CI (−3.48, 0.26)
PF_A-U	0.92 CI (0.80, 0.97) *	0.92 CI (0.81, 0.97) *	0.73 CI (0.33, 0.89) *
PF_r-c	0.82 CI (0.57, 0.93) *	0.82 CI (0.56, 0.93) *	0.55 CI (−0.12, 0.82)
CE_A-U	0.99 CI (0.97, 0.99) *	0.93 CI (0.82, 0.97) *	0.88 CI (0.69, 0.95) *
CE_r-s	0.89 CI (0.74, 0.96) *	0.85 CI (0.64, 0.94) *	0.69 CI (0.24, 0.87) *
SA_A-U	0.96 CI (0.91, 0.98) *	0.96 CI (0.90, 0.98) *	0.78 CI (0.45, 0.91) *
SA_r-s	0.86 CI (0.65, 0.94) *	0.80 CI (0.51, 0.92) *	0.68 CI (0.21, 0.87) *
DS_A-U	0.99 CI (0.97, 0.99) *	0.97 CI (0.92, 0.99) *	−0.37 CI (−3.10, 0.54)
DS_r-s	0.98 CI (0.96, 0.99) *	0.96 CI (0.90, 0.98) *	−0.17 CI (−2.48, 0.61)

Values are intraclass correlation coefficient (ICC) value, 95% confidence interval (CI). * *p* < 0.05. Abbreviations: BH, brow height; A-U, difference between affected and unaffected side; r-c, difference between resting and closed eye state; PF, palpebral fissure; CE, commissure excursion; r-s, difference between resting and smile state; SA, smile angle; DS, dental show.

**Table 3:** Relationship between the Emotrics data and House–Brackmann facial palsy scale.

Characteristics	Correlation Coefficient with HB_E *	Correlation Coefficient with HB_M *	*p*-Value
BH_A-U	−0.07	-	0.75
BH_r-c	0.81	-	<0.01
PF_A-U	0.28	-	0.22
PF_r-c	−0.64	-	<0.01
CE_A-U	-	−0.26	0.25
CE_r-s	-	−0.10	0.66
SA_A-U	-	−0.18	0.44
SA_r-s	-	−0.20	0.39
DS_A-U	-	−0.40	0.11
DS_r-s	-	0.10	0.71

* Spearman’s correlation. Abbreviations: HB_E, House–Brackmann facial palsy scale of eye; HB_M, House–Brackmann facial palsy scale of mouth; BH, brow height; A-U, difference between affected and unaffected side; r-c, difference between resting and closed eye state; PF, palpebral fissure; CE, commissure excursion; r-s, difference between resting and smile state; SA, smile angle; DS, dental show.

**Table 4:** Relationship between the Emotrics data and House–Brackmann facial palsy scale (peripheral facial palsy group).

Characteristics	Correlation Coefficient with HB_E * (*n* = 14)	Correlation Coefficient with HB_M * (*n* = 14)	*p*-Value
BH_A-U	−0.17	-	0.62
BH_r-c	0.92	-	<0.01
PF_A-U	0.43	-	0.19
PF_r-c	−0.77	-	<0.01
CE_A-U	-	−0.26	0.24
CE_r-s	-	−0.17	0.62
SA_A-U	-	−0.18	0.59
SA_r-s	-	−0.50	0.12
DS_A-U	-	−0.41	0.21
DS_r-s	-	0.32	0.34

* Spearman’s correlation. Abbreviations: HB_E, House–Brackmann facial palsy scale of eye; HB_M, House–Brackmann facial palsy scale of mouth; BH, brow height; A-U, difference between affected and unaffected side; r-c, difference between resting and closed eye state; PF, palpebral fissure; CE, commissure excursion; r-s, difference between resting and smile state; SA, smile angle; DS, dental show.

## Data Availability

The data that support the findings of this study are available from the corresponding author, D.Y.K., upon reasonable request.
